# *Rickettsia parkeri* in *Amblyomma maculatum* Ticks, North Carolina, USA, 2009–2010

**DOI:** 10.3201/eid1712.110789

**Published:** 2011-12

**Authors:** Andrea S. Varela-Stokes, Christopher D. Paddock, Barry Engber, Marcee Toliver

**Affiliations:** Mississippi State University, Starkville, Mississippi, USA (A.S. Varela-Stokes);; Centers for Disease Control and Prevention, Atlanta, Georgia, USA (C.D. Paddock);; North Carolina Department of Environment and Natural Resources, Raleigh, North Carolina, USA (B. Engber, M. Toliver)

**Keywords:** Spotted fever group rickettsiae, Rickettsia, vector-borne infections, Rocky Mountain spotted fever, North Carolina, ticks, Candidatus Rickettsia andeanae, Rickettsia parkeri, Amblyomma maculatum

## Abstract

We detected *Rickettsia parkeri* in 20%−33% of *Amblyomma maculatum* ticks sampled in North Carolina. Results highlight the high frequencies of *R. parkeri*–infected ticks in the state with the highest annual incidence of Rocky Mountain spotted fever. Epidemiologic studies are needed to definitively link *R. parkeri* to cases of spotted fever rickettsiosis.

North Carolina historically reports some of the highest annual case counts of Rocky Mountain spotted fever (RMSF) and has accounted for >20% of total cases reported in the United States during the past 30 years (*1*–*4*). However, a species-specific diagnosis directly implicating infection with *Rickettsia rickettsii* is obtained for <10% of reported US cases. In 2010, the Centers for Disease Control and Prevention and Council of State and Territorial Epidemiologists modified the RMSF case designation to spotted fever rickettsiosis, acknowledging the complex epidemiology of tick-borne rickettsioses (*5*). Currently, *R. parkeri* is the only other tick-borne spotted fever group *Rickettsia* (SFGR) species known to cause disease in the southeastern United States, with >30 recognized cases from at least 9 states, including North Carolina (*6*). *R. parkeri* is detected in 20%−43% of *Amblyomma maculatum* ticks from the southeast, far greater than the recognized occurrence of *R. rickettsii* in any other tick species (*6*–*8*). We surveyed *A. maculatum* ticks collected from North Carolina for evidence of *R. parkeri* infection to assess the possibility that SFGR other than *R. rickettsii* result in cases categorized as RMSF in this state.

## The Study

During May–September 2009 and 2010, adult ticks were collected by the Public Health Pest Management Section of the North Carolina Department of Environment and Natural Resources. Large numbers of *A. maculatum* ticks submitted by the general public through a tick-attachment project (www.deh.enr.state.nc.us/phpm/ticks_projects.htm) prompted further investigation in 3 counties in 2010. A total of 234 *A. maculatum* ticks were collected from 27 counties primarily distributed in the coastal plain and piedmont regions of North Carolina (Figure). These included 36 (2009) and 27 (2010) from the attachment project, all of which were removed from humans, except 6 (2009) that were removed from domestic dogs. The remaining 34 (2009) and 137 (2010) specimens were collected by dragging/flagging. Thirteen archived adult *A. maculatum* ticks collected during 1982–2008 were additionally tested, of which 6 were removed from humans. Nine adult *Dermacentor variabilis* ticks from the attachment project and 45 collected at sites with *A. maculatum* ticks were also tested. All ticks were identified by using standard taxonomic keys and were stored in 95% ethanol.

**Figure:**
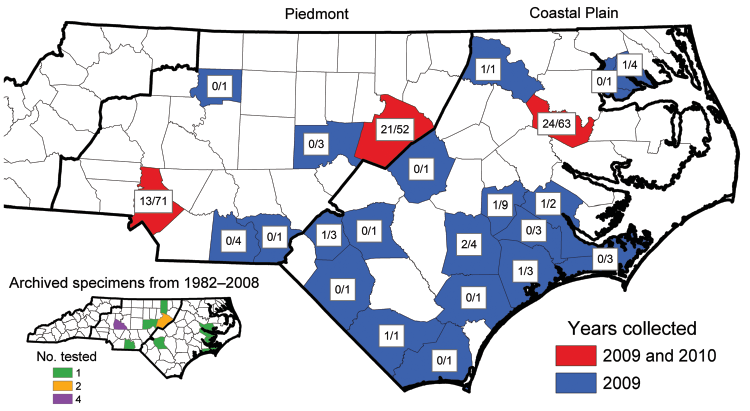
Distribution of *Rickettsia parkeri*–infected *Amblyomma maculatum* ticks collected in North Carolina, USA, during 2009−2010 and in archived specimens (inset). Numbers indicate total number positive for *R. parkeri* by PCR/total number tested in that county.

DNA was extracted by using the QIAmp DNA Mini Kit (QIAGEN, Valencia, CA, USA) for ticks collected in 2009 and Illustra Tissue and Cells genomicPrep Mini Spin Kit (GE Healthcare, Piscataway, NJ, USA) for ticks collected in 2010 and all archived *A. maculatum* and *D. variabilis*. All samples were tested by a PCR targeting a rickettsial outer membrane protein A (*ompA*) gene fragment. *D. variabilis* ticks were tested by using a broad-range SFGR-nested PCR (*9*). *A. maculatum* ticks were tested by using primers specific for *R. parkeri* and *Candidatus* Rickettsia andeanae. *R. parkeri*–specific primers were designed by aligning representative *ompA* gene sequences in GenBank for *R. parkeri*, *R. rickettsii*, *R. peacockii*, *R. amblyommii*, and *Candidatus* R. andeanae and identifying nonconserved regions among sequences. Primers RpompAF (5′-AATGCAGCATTTAGTGATGATGTTAA-3′) and RpompAR (5′-TCCTCCATTTATATTGCCTG-3′) were chosen. Final reagent concentrations were 300 nmol/L for each primer, 1.25 units GoTaq (Promega, Madison, WI, USA), 1.5 mmol/L MgCl_2_, and 200 nmol/L each dNTP. Thermal cycler conditions were as follows: 94°C (2 min); 40 cycles of 94°C (30 s), 54°C (60 s), and 72°C (90 s); and a final extension of 72°C (5 min) to amplify the 447-bp fragment. We confirmed that *R. parkeri*–specific primers would not amplify *R. amblyommii* by testing 6 *A. americanum* ticks infected with *R. amblyommii* (determined by sequencing 17-kDa antigen gene amplicon) because this species has been detected in *A. maculatum* ticks (*10*). To detect *Candidatus* R. andeanae, primers Rx-190-F and Rx-190-R were used in a conventional PCR (*6*). All rickettsial PCRs included a positive control of DNA from cultured *R. parkeri* (Tate’s Hell strain) or a previously confirmed *Candidatus* R. andeanae–infected *A. maculatum* tick, and water controls. DNA extractions, PCRs, and electrophoresis were performed in separate rooms or designated laboratory areas. DNA extractions from archived *A. maculatum* ticks were tested by PCR of a tick mitochondrial 16S rRNA gene amplicon (*11*) to ensure amplifiable DNA. Selected PCR products were submitted to Eurofins MWG Operon (Huntsville, AL, USA). Consensus sequences determined by ClustalX2 alignment for each sample were compared with sequences in GenBank for identification by using a BLAST search (www.ncbi.nlm.nih.gov/blast/Blast.cgi).

An additional 21 *A. maculatum* ticks from Mecklenburg County (2010) were processed to isolate *R. parkeri* as described (*6*). The identity of each isolate was confirmed by *ompA* PCR and sequence analysis.

DNA extracts from 8 female and 6 male ticks (20%) of 70 *A. maculatum* ticks collected in 2009 tested positive for *R. parkeri* (Table). Of these, 6 were collected by dragging or flagging, 2 were unattached on domestic dogs, 5 were crawling or attached on persons, and 1 was on a vehicle. Sequences from 3 *R. parkeri*–positive extracts from 3 different counties were 100% identical to *R. parkeri*; next closest in identity (98%) were *R. sibirica* and *R. africae*. In 2010, 54/164 (27 females; 27 males) (33%) *A. maculatum* specimens tested positive for *R. parkeri*. Sequences from 17 positive samples (3 from Wake County, 6 from Mecklenburg County, 8 from Martin County) were 100% identical to *R. parkeri*. Ten of the 2010 *R. parkeri*–positive ticks were attached to persons; clinical symptoms for these persons were not assessed.

**Table:** *Rickettsia* species detected by PCR in adult *Amblyomma maculatum* ticks collected in North Carolina, USA, 2009−2010

Year, county	No. adult ticks tested (no. found on person or domestic animal)	% Positive	
		*R. parkeri*	*Candidatus* Rickettsia andeanae
2009	70	14 (20)	1 (1)
Anson	4 (0)	0	0
Brunswick	1 (0)	0	0
Carteret	3 (67)	0	0
Chatham	3 (0)	0	0
Chowan	1 (0)	0	0
Columbus	1 (0)	1	0
Craven	2 (50)	1* (50)	0
Cumberland	1 (0)	0	0
Duplin	4 (25)	2 (50)	0
Forsyth	1 (100)	0	0
Halifax	1 (0)	1 (100)	0
Hoke	3 (0)	1 (33)	0
Johnston	1 (0)	0	0
Jones	3 (67)	1 (33)	0
Lenoir	9 (100)	1 (11)	0
Martin	6 (100)	2 (33)	1* (17)
Mecklenburg	1 (0)	0	0
Onslow	3 (33†)	1† (33)	0
Pender	1 (0)	0	0
Perquimans	4 (100)	1* (25)	0
Richmond	1 (100)	0	0
Robeson	1 (0)	0	0
Wake	15 (47)	2‡ (13)	0
2010	164	54 (33)	8 (5)
Martin	57 (46)	22§ (39)	3 (5)
Mecklenburg	70 (3)	13*¶ (17)	5 (7)
Wake	37 (3)	19 (51)	0

*One specimen on person. †Specimen was found on vehicle. ‡Specimens were removed from domestic animals. §Nine specimens were removed from individual persons. ¶Thirteen specimens were processed for culture isolation; 3 isolates obtained.

*Candidatus* R. andeanae was detected in 9 tick extracts. Sequences of all 2010 positive ticks were 100% identical to GenBank *ompA* sequences for *Candidatus* R. andeanae. One male *Candidatus* R. andeanae–positive tick from Martin County was co-infected with *R. parkeri*, which was confirmed by sequencing. No archived *A. maculatum* ticks were positive by PCR for *Candidatus* R. andeanae or *R. parkeri*. However, 8 archived samples showed faintly staining bands for mitochondrial 16S rRNA gene amplicons, suggesting loss of DNA integrity in these older samples. Three isolates of *R. parkeri*, designated NC-3, NC-8, and NC-15, were obtained in Vero E6 cell cultures. No SFGR was detected by PCR in any *D. variabilis* ticks.

## Conclusions

Until recently, *A. maculatum* was considered an incidental tick species in North Carolina (*12*,*13*); however, we identified an overall prevalence of *R. parkeri* in 29% of *A. maculatum* ticks from multiple sites in North Carolina considered endemic for RMSF. These data, coupled with the frequency of *R. parkeri*–positive ticks removed from humans, suggest that *A. maculatum* ticks are well established in North Carolina and that *R. parkeri* causes at least some cases of spotted fever rickettsiosis in this state. We examined a small number of *D. variabilis* ticks; however, none were infected with *R. rickettsii*, consistent with previous surveys of this tick for SFGR in North Carolina and other states (*8*,*14*,*15*). More extensive surveys of *D. variabilis* may be warranted to better determine the relative contribution of this tick to spotted fever rickettsiosis in North Carolina. The pathogenicity and clinical significance of *Candidatus* R. andeanae are unknown; however, this rickettsia is detected in *A. maculatum* ticks less frequently than *R. parkeri* and thus far has not been directly associated with human illness (*6*). *Candidatus* R. andeanae has been detected in singly infected *A. maculatum* ticks from Virginia (*7*), Florida, Mississippi, and Georgia (*9*) but to our knowledge has not been found in ticks co-infected with other rickettsiae. Further studies that causally link *R. parkeri* in *A. maculatum* ticks with human disease in North Carolina are necessary to incriminate it as a causative agent in this state.
